# Retraction: CircBANP acts as a sponge of let-7a to promote gastric cancer progression *via* the FZD5/Wnt/β-catenin pathway

**DOI:** 10.1039/d1ra90064a

**Published:** 2021-02-01

**Authors:** Laura Fisher

**Affiliations:** Royal Society of Chemistry Thomas Graham House, Science Park, Milton Road Cambridge CB4 0WF UK advances-rsc@rsc.org

## Abstract

Retraction of ‘CircBANP acts as a sponge of let-7a to promote gastric cancer progression *via* the FZD5/Wnt/β-catenin pathway’ by Jin Xun *et al.*, *RSC Adv.*, 2020, **10**, 7221–7231, DOI: 10.1039/C9RA09887A.

The Royal Society of Chemistry hereby wholly retracts this *RSC Advances* article due to concerns with the reliability of the data. The images in the article, and the raw data provided by the authors, were screened by an image integrity expert. Many of the backgrounds to the western blot panels in Fig. 5E and H have repeating features, indicating they have been manipulated. The individual bands do not appear genuine and may have been added onto an empty background.

Analysis of the raw data provided by the authors showed that the backgrounds of many of the panels in the raw data of Fig. 5E and H do not match the backgrounds in the figure panels. Furthermore, some of the raw data provided for Fig. 5E did not match the corresponding published data.

For each of the cyclin D1 and p-β-catenin panels in Fig. 6D, only one of the bands matched the raw data. For both panels, the backgrounds did not match.

Given the significance of the concerns about the validity of both the data in the article and the raw data provided by the authors, the findings presented in this paper are not reliable.

Lianfeng Zhang opposes the retraction. The other authors have been informed but have not responded to any correspondence regarding the retraction.

Signed: Laura Fisher, Executive Editor, *RSC Advances*

Date: 19^th^ January 2021

## Supplementary Material

